# Impact of COVID-19 on Thrombus Burden and Outcome in Acute Myocardial Infarction

**DOI:** 10.7759/cureus.16817

**Published:** 2021-08-01

**Authors:** Bhagya N Pandit, Abhinav Shrivastava, Ranjit K Nath, Dheerendra Kuber, Santosh K Sinha, Puneet Aggarwal

**Affiliations:** 1 Cardiology, Atal Bihari Vajpayee Institute of Medical Sciences (ABVIMS) and Dr Ram Manohar Lohia (RML) Hospital, New Delhi, IND; 2 Cardiology, Laxmipat Singhania (LPS) Institute of Cardiology, Kanpur, UP, IND

**Keywords:** covid-19, acute myocardial infarction, percutaneous coronary intervention, coronary thrombus, st-elevation myocardial infarction (stemi)

## Abstract

Background

Cardiovascular manifestations are one of the most common complications in coronavirus disease 2019 (COVID-19) infection and are associated with increased mortality. However, the impact of COVID-19 infection on thrombus burden and the outcome of acute myocardial infarction (AMI) has not been studied.

Methods

This was a retrospective, observational study that included all adult patients (>18 years) diagnosed with AMI with or without COVID-19 infection. Epidemiological, laboratory, clinical, interventional, and outcome data were extracted and the impact of COVID-19 on thrombus burden and the primary clinical composite endpoint of all-cause death during hospital admission or 30 days after discharge was studied.

Results

The study population included 336 patients, including 56 patients with COVID and AMI and 280 patients with AMI without COVID-19 infection. Chest pain was the most common symptom (84.8%) while one or more co-morbidity was present in 117 (34.8%) patients. Forty-eight patients in the AMI with COVID group had ST-segment elevation myocardial infarction (STEMI) while 256 patients in the AMI without COVID group had STEMI, eight patients in the AMI with COVID group had non-ST-segment elevation myocardial infarction (NSTEMI), and 24 in the AMI without COVID group had NSTEMI. Patients with COVID-19 co-infection had a higher thrombus burden as compared to the patients without COVID-19 AMI group (p-value 0.008). The primary outcome in the form of all-cause mortality was seen in 13 (3.9%) patients, which was also more in the AMI with COVID group.

Conclusion

COVID-19 in AMI is a state of high thrombus burden associated with higher mortality, especially in patients with chronic co-morbidities.

## Introduction

Coronavirus disease 2019 (COVID-19) infection in the majority of patients presents as a mild disease. However, a strong relationship has been established between the presence of myocardial injury and poor outcomes in COVID-19. Those with critical illness demonstrate elevated cardiac biomarkers and rising levels have correlated to poorer clinical outcomes [1-2]. Potential mechanisms causing myocardial injury may involve angiotensin-converting enzyme (ACE)-2 expression within the myocardium and coronary vessels triggering local inflammation, hypercoagulopathy, and thrombosis. The direct activation of platelets by severe acute respiratory syndrome coronavirus 2 (SARS-CoV-2) also likely generates a pro-thrombotic milieu [3]. The resulting hypercoagulability in patients may result in coronary thrombosis and cause acute myocardial infarction (AMI) in the form of type I myocardial infarction (MI). MI can also be precipitated due to cytokine-induced atherosclerotic plaque instability and rupture or non-plaque thrombosis. Inflammatory overdrive and hypoxia have also been shown to induce abnormalities of coagulation [4]. Indeed, patients with co-existing COVID-19 and AMI have significantly more rates of thromboembolic complications and higher thrombus grade post percutaneous coronary intervention [5]. Type II MI may result from an ongoing supply/demand mismatch due to respiratory failure and hypoxia.

In view of the grim pathophysiological processes involved in an AMI in a COVID-19 positive patient, he/she should have different clinical outcomes when compared to AMI in a non-COVID infected patient. Despite the increasing literature regarding the COVID-19 positive population, there is limited information regarding clinical characteristics and outcomes of patients with AMI in COVID-19 co-infection when compared to AMI in a normal population. The impact of the COVID-19 has been shown to increase the case-fatality rates in patients with AMI and active COVID 19 infection; however, limited studies regarding outcome have been done with a reported mortality of around 27.3% [6-7]. The current study aims to find the impact of COVID-19 on thrombus burden and the outcome of AMI in COVID-19 patients as compared to non-COVID patients at a tertiary care center in a developing country.

## Materials and methods

Study design and participants

Our study was a retrospective, observational study including all adult patients (>18 years) presenting to the emergency services of our hospital and diagnosed with AMI as per the fourth universal definition of MI [8] and screened for SARS-CoV-2/COVID-19 via the SARS-CoV-2 reverse transcriptase-polymerase chain reaction (RT-PCR) examination between March 24, 2020, and March 31, 2021. The patients were followed up during the hospital stay and for one month post-discharge.

Data collection

Epidemiological, laboratory, clinical management, and outcome data were extracted from all the enrolled patients after due consent from them. Two physicians checked the data, and a third researcher adjudicated any difference in interpretation between the two primary reviewers. Approval from the local institutional ethical committee was taken, and it conforms to standards currently applied in the country of origin.

Treatment strategies

The patients enrolled with AMI were divided into those SARS-CoV-2 RT-PCR-positive (labeled as AMI with COVID) and SARS-CoV-2 RT-PCR-negative (labeled as AMI without COVID). The patients enrolled with AMI were divided into those presenting with ST-segment elevation myocardial infarction (STEMI) and non-ST-segment elevation MI (NSTEMI). In patients with confirmed STEMI, the patient was taken up for either immediate primary percutaneous coronary intervention (PCI) (depending upon the availability of a catheterization laboratory and personnel) or immediate fibrinolysis was performed within 30 minutes of first medical contact if there were no contraindications. The need for rescue intervention was assessed at 90 minutes post fibrinolysis. Urgent angiography was performed in patients with 1) hemodynamic instability, 2) refractory ventricular arrhythmias, 3) ongoing ischemic chest pain, or 4) <50% resolution of the ST-segment elevation in the worst lead. Other patients were monitored for 48-72 hours and scheduled for routine cardiac catheterization non-urgently.

In patients with NSTEMI, the patient's risk assessment was done with the GRACE (Global Registry of Acute Coronary Events) score, and urgent angiography was done if the GRACE score was more than 140 or he belonged to very high risk or high risk as per the European Society of Cardiology (ESC) guidelines [9]. All patients were started on guideline-directed medical therapy at diagnosis of AMI as clinically permissible. Stable patients were taken up for routine coronary angiography.

Outcome

The primary clinical composite endpoint in this study was an occurrence of all-cause mortality during hospital admission or 30 days of hospital discharge on telephonic consultation.

Laboratory procedures

Electrocardiogram (ECG) was performed on all patients before diagnosis and then at half-hourly intervals post thrombolysis or any intervention for at least four hours and then afterward whenever clinically indicated. Troponin T and creatine kinase-MB (CK- MB) were done in all patients at presentation. Routine blood examinations were done, including complete blood count, arterial blood gases, serum biochemical tests (including serum electrolytes, renal and liver function tests). Chest radiographs were also done for all patients. Nasal and oropharyngeal swabs were taken from all patients in our study and tested to detect COVID-19 using RT-PCR for confirmation. Specimens were obtained for a SARS-CoV-2 RT-PCR examination on the day of admission and again on Day 5 of admission in case the first sample was negative. A single positive test was sufficient to declare positive results. Two consecutive negative tests were done for patients with a highly suspicious radiological appearance on chest X-ray before being labeled COVID-19 negative. The treating physician determined the frequency of examinations.

Definitions used

AMI was defined as per the 4th Universal definition of MI [8]. ST-elevation on electrocardiogram (ECG) was defined as new or presumed new, J-point elevation ≥1 mm (1 mm = 0.1 mV) in all leads other than V2 and V3 where the following cut-points apply: ≥2 mm in men ≥40 years; ≥2.5 mm in men <40 years; or ≥1.5 mm in women regardless of age or new or presumably new left bundle-branch block on the 12-lead ECG. ST depression and T wave changes were defined as new or presumed new horizontal or downsloping ST-depression ≥0.5 mm in two contiguous leads and/or T inversion >1 mm in two contiguous leads with a prominent R wave or R/S ratio >1. MI was defined as STEMI or NSTEMI when biomarkers CK-MB or troponin T was elevated in addition to the ECG changes.

Time to treatment was defined from symptom onset to the first medical contact in our hospital. Primary percutaneous coronary intervention (PPCI) was defined as PCI done within 12 hours of symptom onset in a patient not receiving fibrinolysis. The time to start of reperfusion therapy was defined as the time to intravenous injection of fibrinolytic or time to wire crossing the culprit lesion in patients treated with fibrinolysis and PPCI. Rescue PCI was defined as PCI mandated by 1) hemodynamic instability, 2) refractory ventricular arrhythmias, 3) ongoing ischemic chest pain, or 4) <50% resolution of the ST-segment elevation in the worst lead within 90 minutes after the administration of fibrinolysis. The pharmaco-invasive strategy was defined as fibrinolysis followed by a rescue or urgent PCI or by routine elective PCI (beyond 3 hours of fibrinolytic administration). Major bleeding was defined as type 3 or type 5 of the Bleeding Academic Research Consortium [10].

Statistical analysis

Continuous variables were expressed as mean ±SD and compared with the student t-test when normally distributed and as median (quartiles) and compared with the Mann-Whitney U test when the data did not fit the normal distribution. Categorical variables were reported as numbers and percentages and compared with the χ2 test. Proportions for categorical variables were compared using the chi-square test. All statistical analyses were performed using SPSS (Statistical Package for the Social Sciences) version 20.0 software (IBM Corp., Armonk, NY). For unadjusted comparisons, a two-sided α of less than 0.05 was considered statistically significant. The analyses have not been adjusted for multiple comparisons, and given the potential for a type I error, the findings should be interpreted as exploratory and descriptive.

## Results

Patient characteristics

The study population included 336 patients who were admitted with an AMI. The baseline characteristics of the patients are as shown in Table 1. The mean age at admission was 60.31 ± 9.1 years, and 121 patients (36%) were female. Out of all patients, 56 (16.7%) tested positive for COVID-19, 32 (9.5%) had NSTEMI, and 304 (89.5%) had STEMI.

**Table 1:** Baseline demographics and clinical characteristics of the study population AMI: acute myocardial infarction; COPD: chronic obstructive pulmonary disease

Characteristics	Total (n=336) N(%)	AMI with COVID (n=56) N(%)	AMI without COVID (n=280) N(%)	P-value
Age	60.31 (±9.1)	61.59 (±8.8)	60.05 (±9.14)	0.25
Male	215 (64%)	33 (15.3%)	182 (84.7%)	0.45
Female	121 (36%)	23 (19%)	98 (81%)	0.45
Cough	28 (8.3%)	7 (2.1%)	21 (7.5%)	0.285
Fever	14 (4.2%)	8 (14.3%)	6 (2.1%)	0.001
Sore throat	11 (3.3%)	4 (7.1%)	7 (2.5%)	0.09
Dyspnoea	60 (17.9%)	11 (19.6%)	49 (17.5%)	0.704
Myalgia and fatigue	29 (8.6%)	8 (14.3%)	21 (7.5%)	0.116
Chest pain	285 (84.8%)	47 (83.9%)	238 (85%)	0.839
Headache	8 (2.4%)	1 (1.8%)	7 (2.1%)	1.0
Loose stools	9 (2.77%)	2 (3.6%)	7 (2.5%)	0.65
Anosmia	12 (3.6%)	10 (10.8%)	2 (0.7%)	0.001
Vomiting	24 (7.1%)	3 (5.4%)	21 (7.5%)	0.778
Co-morbidities (at least 1)	117 (34.8%)	33 (58.9 %)	84 (30%)	0.001
Diabetes mellitus	40 (11.9%)	12 (21.4%)	28 (10%)	0.023
Hypertension	53 (15.8%)	18 (32.1%)	35 (12.5%)	0.001
Coronary artery disease	16 (4.8%)	2 (3.6 %)	14 (5%)	1.0
Cerebrovascular accident	8 (2.4%)	1 (1.8%)	7 (2.5%)	1.0
COPD	43 (12.8%)	8 (14.3%)	35 (12.5%)	0.66
Hypothyroid	10 (3%)	3 (5.4%)	7 (2.5%)	1.0

Chest pain was the most common symptom in both AMI with COVID and AMI without COVID patients, which was present in 85% of patients at presentation. Fever was seen significantly more in AMI with COVID patients. Generalized fatigue and upper respiratory tract infection symptoms tended to be more in the AMI with COVID group. Of the 336 patients, 117 patients had one or more co-morbidities with AMI with COVID patients having significantly more co-morbidities (58.9% vs 30%). Hypertension (32.1% vs 12.5%) and diabetes mellitus (40; 11.9%) were the most common co-existing illness and were significantly more in the AMI with COVID group.

Laboratory findings

The laboratory parameters and clinical characteristics of the patients on admission were as shown in Table 2. In laboratory parameters, hypoalbuminemia was present in 152 (45.2%) patients on admission and was the most common finding. Anemia was present in 143 (42.6%) patients and leucopenia was present in 27 (8%) patients. Thrombocytopenia was seen in 29 (8.6%) and pancytopenia in 10 (3%) patients. Twenty-six (7.7%) patients developed acute kidney injury. Alanine aminotransferase, aspartate aminotransferase, and serum bilirubin were elevated less commonly.

**Table 2:** Baseline laboratory parameters of the study population AMI: acute myocardial infarction; LVEF: left ventricular ejection fraction; AST: aspartate aminotransferase; ALT: alanine aminotransferase; CK-MB: creatine kinase-MB

Characteristics	Total (n=336) Mean ±SD	AMI with COVID (n=56) Mean ±SD	AMI without COVID (n=280) Mean ±SD	P-value
LVEF (%)	40.06 (±4.9)	39.11 (±4.96)	40.25 (±4.87)	0.11
Hemoglobin	12.59 (±1.46)	12.53(±1.51)	12.61 (±1.45)	0.72
Total leucocyte count	6936 (±2631)	6505 (±2718)	7022 (±2610)	0.18
Platelets	1.88 (±0.55)	1.90 (±0.55)	1.88 (±0.50)	0.69
AST	38 (±9.4)	39.54 (±4.1)	37.6(±4.2)	0.96
ALT	33 (±11.4)	34.8 (±3.8)	33.6 (±3.9)	0.87
Bilirubin	0.74 (±0.25)	0.75 (±0.25)	0.74 (±0.24)	0.77
Urea	33.3 (±11.4)	34.73 (±13.9)	33.38 (±10.8)	0.42
Creatinine	0.874 (±0.29)	0.88 (±0.29)	0.84 (±0.29)	0.36
Albumin	3.5 (±0.40)	3.5 (±0.38)	3.5 (±0.40)	0.89
CK MB	146 (±27.4)	147 (±26.04)	145 (±28)	0.65

Treatment and main interventions

Treatment and intervention data of the patient was as shown in Table 3. Three hundred thirty-six patients of AMI were followed up in the study. Fifty six (16.7%) patients were COVID-19 positive. Out of 304 patients who had presented with STEMI, 251 (82.6%) patients presented in the window period. Seventy-three (29%) were taken up for primary PCI and 178 (70.9%) were fibrinolyzed upfront. Of the primary PCI group, 15 (31.25%) patients were COVID-19 positive and 58 (22.6%) patients were COVID 19 negative. Out of the 178 patients who were fibrinolyzed, 24 were COVID-19 positive and 154 were AMI without COVID. Successful fibrinolysis was done in 22 (91.3%) AMI with COVID patients and in 129 (83.7%) AMI without COVID patients. There was no significant difference in the rates of success of fibrinolysis. Thirty-one (15.4%) were taken up for rescue PCI of whom five patients were from the AMI with COVID group. Ninety-seven (48.3%) patients were taken up for elective PCI from the post-fibrinolysis/late presentation patients of whom five patients were from the AMI with COVID group and 92 were from the AMI without COVID group. Of the 201 patients who underwent coronary angiography, five (2.5%) were finally diagnosed with type 2 AMI with non-obstructive coronary arteries; three patients were COVID-19 positive, whereas two were COVID-19 negative, the difference between the two being non-significant.

**Table 3:** Treatment and intervention data of study population AMI: acute myocardial infarction; STEMI: ST-segment elevation myocardial infarction; NSTEMI: non-ST-segment elevation myocardial infarction: AWMI: anterior wall myocardial infarction; ALWMI: anterolateral wall myocardial infarction; IWMI: inferior wall myocardial infarction; LWMI: lateral wall myocardial infarction; PWMI: posterior wall myocardial infarction; PPCI: primary percutaneous coronary intervention; PCI: percutaneous coronary intervention: PTCA: percutaneous transluminal coronary angioplasty; CAG: coronary angiography; LAD: left anterior descending artery; LM: left main coronary artery; LCx: left circumflex artery: RCA: right coronary artery

Characteristics	Total (n=336) N(%)	AMI with COVID (n=56) N(%)	AMI without COVID (n=280) N(%)	P-value
STEMI	304 (90.5%)	48 (85.7%)	256 (91.4%)	0.2
NSTEMI	32 (9.5%)	8 (14.3%)	24 (8.6%)	0.2
AWMI/ALWMI	148 (48.68%)	27 (56.25%)	121 (47.3%)	0.556
IWMI	143 (47%)	21 (43.75%)	122 (47.6%)	0.46
LWMI	6 (1.97%)	0	6 (2.3%)	0.59
PWMI	7 (2.3%)	0	7 (2.7%)	0.61
Late Presentation	53 (17.4%)	9 (17.9%)	44 (15.7%)	0.692
Fibrinolysis	178 (70.9%)	24 (60.5%)	154 (72.6%)	0.078
Successful fibrinolysis	151 (84.8%)	22 (91.3%)	129 (83.7%)	0.30
Unsuccessful fibrinolysis	27 (15.2%)	2 (8.7%)	25 (16.2%)	0.3
Tenecteplase	45 (25.4%)	9 (39%)	36 (23.4%)	0.67
Streptokinase	133 (74.6%)	15 (60.86%)	118 (76.6%)	0.025
PPCI	73 (29%)	15 (31.25%)	58 (22.6%)	0.37
PCI done	201 (59.8%)	25 (44.6%)	176 (62.8%)	0.143
Rescue PCI	31 (15.4%)	5 (20%)	26 (14.8%)	0.218
Elective PCI	97 (48.3%)	5 (20%)	92 (52.2%)	0.001
PTCA	196 (97.5%)	22 (88%)	174 (98.8%)	0.21
Non-obstructive coronary artery disease	5 (2.5%)	3 (12%)	2 (1.13%)	0.32
LAD	107 (54.6%)	15 (68%)	92 (52.9%)	0.43
LCx	39 (19.9%)	3 (13.64%)	36 (20.7%)	0.115
RCA	50 (25.5%)	4 (18.18%)	46 (26.4%)	0.032

Percutaneous transluminal coronary angioplasty was performed in 196 patients, 22 from the AMI with COVID group, and 174 from the AMI without COVID group. We found no significant differences in the anatomical characteristics of the culprit vessel or the number of implanted stents in the patients in both groups.

Thrombus burden and outcomes

The modified thrombus grade for cases with thrombus grade 4/5 was significantly higher in the AMI with COVID group (modified thrombus grade 4/5 in 32% vs 8%, p = 0.002) as shown in Table 4. There was significantly greater use of aspiration thrombectomy (12% vs 0.5%) (p = 0.01) in patients with AMI with COVID. However, thrombolysis in myocardial infarction (TIMI) flow grade 3 was achieved at similar high levels in both groups. Stent thrombosis was present significantly more in the AMI with COVID group [3 (12%) vs 1 (0.5%)].

**Table 4:** Thrombus burden and outcome data of study population AMI: acute myocardial infarction

Characteristics		Total (n= 336) N(%)	AMI with COVID (n=56) N(%)	AMI without COVID (n=280) N(%)	P-value
Modified thrombus grade	0	29 (14.4%)	2 (8%)	27 (15.34%)	0.008
	1	47 (23.4%)	4 (16%)	43 (24.4%)	
	2	78 (38.8%)	7 (28%)	71 (40.34%)	
	3	25 (12.4%)	4 (16%)	21 (12%)	
	4	16 (8%)	5 (20%)	11 (6.2%)	
	5	6 (3%)	3 (12%)	3 (1.7%)	
Modified thrombus grade 4-5		22 (10.9%)	8 (32%)	14 (8%)	0.002
Thrombosuction Used		20 (9.9%)	8 (32%)	12 (6.8%)	0.001
Stent thrombosis		4 (2%)	3 (12%)	1 (0.5%)	0.01
Major bleed		1 (1.8%)	1 (100%)	0	1.0
Mortality		13 (3.9%)	7 (12.5%)	6 (2.1%)	0.002

Thirteen (3.9%) patients met the primary outcome of all-cause mortality. The AMI with COVID group had significantly higher mortality [7 (12.5%) vs 6 (2.1%)] than the AMI without COVID group.

## Discussion

This observational study represents comparative data to describe the impact of COVID-19 infection on patients presenting with AMI in tertiary care, primary PCI-enabled centers. The optimal strategy for the management of patients with AMI in the current COVID-19 pandemic presents a greater challenge than from the pre-pandemic era. The patients in the study were followed up for the duration of their hospital stay and one month after discharge for the primary outcome, that is, all-cause mortality. The main finding of the study was of increased in-hospital mortality in AMI patients infected with COVID-19. This analysis also demonstrates a clear signal of increased thrombus burden in AMI patients who are infected with COVID-19 compared with patients who are not.

Male predominance has been demonstrated in the majority of the studies. COVID-19 has so far shown sex-biased mortality, with a higher death rate in males. This difference could be attributed in part to the differential effect of testosterone and estrogen on the immune system. Females having two X chromosomes possess a higher number of immune-related genes, which provides a stronger immune response. The robust immune system in females has been shown to regulate viral infections and lower mortality [11]. Although males were the more prevalent group in our study, there was no difference in mortality outcomes between the two sexes.

Chest pain was the most common presenting feature in the study group. However, fever was seen more AMI with COVID patients at presentation [8 (14.3%) vs 6 (2.1%)] (p-value 0.001). Patients with COVID-19 more frequently had atypical symptoms, particularly those suggestive of a respiratory infection. Similar findings have also been previously reported [12].

Patients with co-morbidities were more likely to end up being infected with COVID-19 (58.9 % vs 30%), p- 0.001). Similar findings have been reported in a previous study where the prevalence of hypertension in AMI patients has been described as high as 80% and diabetes mellitus in 27% of patients with COVID-19. Also, the prevalence of hypertension seems to be higher in COVID-19 patients who develop severe disease versus those who do not [13]. The mechanisms underlying potential relationships between hypertension and COVID-19 are not known but the key role of the renin-angiotensin-aldosterone system (RAAS)/ACE2 in the pathophysiology of hypertension makes it is possible that any dysregulation of this system may be important [14]. Diabetic patients suffer from a less robust immune system due to chronic hyperglycemic and inflammatory states, and hyperglycemia is an important risk factor for COVID-19 progression and death.

There is a clear signal of increased thrombus burden in AMI patients who are infected with COVID-19 compared with AMI patients who are not. This is evidenced by a higher incidence of thrombotic culprit lesions as well as stent thrombosis, higher thrombus grade, and associated increased use of thrombus aspiration in patients of AMI infected with COVID-19. COVID-19 infection is associated with a prothrombotic state. Venous thromboembolic complications, both clinically apparent and subclinical, have been an important manifestation of the disease and one that is related to disease severity and outcome [15-16]. Higher thrombus burden has been associated with COVID-19 patients with AMI in previous studies, suggesting a question of more aggressive antithrombotic therapy in selected COVID-19 STEMI cases [5].

COVID-19-related pathophysiology causing AMI events are unknown but might be caused due to acute plaque rupture or erosion facilitated by systemic inflammation, microvascular thrombosis due to hypercoagulability, and/or endothelial dysfunction [17]. The latter is known to play a vital role in arterial hypertension and thrombosis and has recently been associated with COVID-19 [5]. Endothelial inflammation in COVID-19 might affect various vascular beds, thereby increasing the susceptibility for thromboembolic and septic complications or multiorgan failure [18]. Thus, myocardial ischemia due to AMI might be even aggravated by COVID-19-induced generalized microvascular dysfunction and systemic vascular damage.

The AMI with COVID group had significantly higher mortality [7 (12.5%) vs 6 (2.1%)] than the AMI without COVID group. Patients in the coronary risk groups of diabetes and hypertension had increased incidence of COVID-19 and subsequent case fatality. Our data point toward higher morbidity and mortality in the COVID-19 group compared with the non-COVID-19 group. The majority of deaths in this study were due to multiorgan dysfunction, indicating systemic vascular damage due to COVID-19 infection [6]. COVID-19 and AMI’s concomitant occurrence might be responsible for the increased mortality seen in our observation

Study limitations

The study is a single-center, retrospective study and might not represent the real impact of COVID-19 on AMI. So, a more extensive multicentered study might bring better insight. All patients with AMI did not undergo coronary angiography at presentation so the amount of thrombus burden might not represent the true thrombus burden in the study population.

## Conclusions

AMI in COVID-19 infection is a state of high thrombus burden associated with higher mortality, especially in patients with chronic co-morbidities. However, a larger study would be required to generalize the results.
